# Anal Involvement in Pemphigus Vularis

**DOI:** 10.1155/2013/609181

**Published:** 2013-11-05

**Authors:** Somayeh Khezri, Hamid-Reza Mahmoudi, Seyedeh Nina Masoom, Maryam Daneshpazhooh, Kamran Balighi, S. Hamed Hosseini, Cheyda Chams-Davatchi

**Affiliations:** ^1^Autoimmune Bullous Diseases Research Center, Department of Dermatology, Tehran University of Medical Sciences, Razi Hospital, Vahdate-Eslami Square, Tehran 11996, Iran; ^2^Knowledge Utilization Research Center, School of Public Health, Tehran University of Medical Sciences, Tehran 1417613151, Iran

## Abstract

*Background*. Pemphigus vulgaris (PV) is an autoimmune blistering disease of the skin and mucosa. Anal mucosa may be involved in PV, but the frequency and clinical profile are not fully ascertained. *Objective*. The aim was to investigate the involvement of the anal area in newly diagnosed PV patients. *Patients and Methods*. A total of 168 consecutive newly diagnosed PV patients were enrolled. Anal symptoms and signs, involvement of other body sites, and severity of disease were recorded. *Results*. A total of 47 out of 168 patients (27.9%) had involvement of the anal area. Anal involvement was significantly associated with PV lesions in ophthalmic (*P* = 0.03), nasal (*P* = 0.02), and genital mucosa (*P* < 0.001) but not the oral cavity (*P* = 0.24). There was a significant association between number of involved mucosal sites and anal involvement (*P* < 0.001). Anal involvement was associated with oral severity (*P* = 0.02). Constipation was the most frequent symptom (73.8%) followed by pain on defecation (50%). Seventeen patients (36%) were symptom-free. Erosion was the most frequent sign (91.5%). *Conclusion*. Anal involvement in PV seems to be more frequent than previously assumed. Routine anal examination is recommended even in asymptomatic patients as anal involvement appears to correlate with the severity of PV.

## 1. Introduction

Pemphigus vulgaris (PV) is a rare, autoimmune, potentially fatal mucocutaneous bullous disease in which pathogenic autoantibodies are directed against the keratinocyte cell surface molecules desmoglein 3 (Dsg3) and to a lesser extent Dsg1 [1]. The incidence of this disease varies from 0.16 to 1.62 cases per 100,000 with increased incidence in Jews, Indians, and middle easterners [2]. PV is characterized by bullae that typically begin in the oral cavity and may spread to involve the skin. Other mucosal surfaces including conjunctiva, nasal mucosa, pharynx, larynx, epiglottis, esophagus, cervix, vagina, and penile mucosa may also be affected in the course of disease [3–8]. Anal involvement may also be seen in PV but its frequency and clinical profile are not fully ascertained yet [9–12]. The aim of this study was to investigate the involvement of the anal area in newly diagnosed PV patients presenting to the Autoimmune Bullous Diseases Research Center (ABDRC), Tehran, Iran, during a 15-month period.

## 2. Patients and Methods

This prospective study included 168 consecutive patients newly diagnosed with PV, attending the ABDRC, between October 2009 and January 2011. The diagnosis of PV was based on the presence of clinical features of the disease, including mucocutaneous bullae and erosions along with histopathological (suprabasal cleft and acantholysis) and direct immunofluorescence (lattice-like intercellular epidermal IgG and/or C3 deposits) findings of the biopsy material [13]. Only patients with new-onset untreated disease were enrolled in this study and all subjects underwent physical examination of the anal area. Anoscopy was not performed.

The following information was collected on each patient: (1) age at onset of PV and gender; (2) anal symptoms and signs; (3) nonanal involvement; (4) severity of disease. The severity of skin and mucosal disease was rated based on a grading system proposed by Harman et al. [14] as follows: oral grading: Grade 0, without any lesion; Grade I, minor activity (up to three lesions); Grade II, moderate activity (more than three but less than 10 erosions or generalized desquamative gingivitis); Grade III, severe (more than 10 discrete erosions or extensive, confluent erosions, or generalized desquamative gingivitis with discrete erosions at other oral sites). Skin grading: Grade 0, without any lesions; Grade I, minor activity (less than five discrete lesions); Grade II, moderate activity (more than five but less than 20 discrete lesions); Grade III, severe (more than 20 discrete lesions or extensive, confluent areas of eroded skin). Only lesions within 2 cm or less from the anal orifice were considered as anal involvement.

Statistical analysis was performed by Student's *t*-test for differences in means of ages of patients with or without anal lesions. The chi-square test was used to analyze differences between involvement of different anatomical sites in patients with or without anal involvement. *P* value <0.05 was considered significant. Fisher's exact test was used wherever necessary.

## 3. Results 

A total of 168 newly diagnosed PV patients were examined, 92 patients (54.8%) were female, and 76 patients (45.2%) were male (M : F ratio = 1 : 1.21). Age distribution of PV was from 19 to 72 years with a mean ± SD of 44 ± 12.6 years. A total of 47 out of 168 patients (27.9%) had involvement of the anal area. The lesions were confined to the stratified epithelium of the anal region.

Table 1 shows characteristics of patients with or without anal involvement. Twenty-three of 47 patients with anal involvement were female (48.9%) and 24 patients (51.1%) were male (M : F ratio= 1 : 0.96). Forty-one (87.2%) out of 47 patients with anal involvement had concomitant oral lesions, while this figure was 79.3% (96 out of 121) for patients without anal involvement. The difference was not significant (*P* = 0.24). On the other hand, anal involvement was significantly associated with PV lesions in other mucosal sites including ophthalmic (*P* = 0.03), nasal (*P* = 0.02), and genital mucosa (*P* < 0.001). 

Focusing on severity of oral disease, 54 cases (32.1%) were grade II followed by grade I (51 cases, 30.4%), grade III (32 cases, 19%), and grade 0 (31 cases, 18.5%). Significant association between severity of oral disease and anal involvement was seen (*P* = 0.02). Skin grading was as follows: 6 cases (3.6%) were grade 0, grade I (39 cases, 23.2%), grade II (56 cases, 33.3%), and grade III (67 cases, 39.9%). There was no association between anal involvement and severity of skin disease (*P* = 0.06).

Two of our patients (4.3%) showed anal lesions in the absence of involvement of other mucosal surfaces; 16 patients (34%) had involvement in one other mucosal site in addition to anal area, and 29 out of 47 patients with anal lesions (62%) showed involvement of PV in at least two other mucosal sites. There was a significant association between the number of involved mucosal sites and anal involvement (*P* < 0.001).

Table 2 shows clinical signs and symptoms reported by patients. Thirty out of 47 patients with anal lesions (64%) complained of anorectal symptoms, while 17 patients (36%) were symptom-free. Constipation was the presenting symptom in the majority of cases (73.8%) followed by pain on defecation (50%). Erosion was found in 43 patients (91.5%) and was the most common anal sign with a mean of 1.3 anal erosions per patient. 

## 4. Discussion

Our study shows greater frequency of anal involvement in PV patients than previous reports (27.9%). Although anal area is a well-known site of involvement in PV, the frequency of this involvement is not fully investigated and figures vary widely (2% [2], 9.3% [10, 11], and 16.5% [15]). There are several reasons for the underestimation of the incidence of anal PV: firstly there may be underreporting of cases, because patients are uncomfortable discussing anal symptoms and may attribute them to other causes such as fissures or hemorrhoids [10, 11]; secondly physicians may not routinely examine the anal area, and at last a significant number of lesions may be asymptomatic or subtle. We performed anal examination regardless of patients' symptoms and severity of disease.

In this study the presence of anal involvement correlated with the severity of mucosal disease as well as with the number of other involved mucosal sites. To our knowledge no study had ascertained anal involvement with the severity of PV. Most of our patients with anal involvement (62%) showed involvement of multiple other mucosal sites. The few patients described in the literature also tended to have involvement of PV at multiple sites, especially the oral mucosa. Epstein et al. reported a patient with widespread involvement at multiple mucosal and cutaneous sites [16]. Malik et al. [17] reported 16 patients with anal involvement and suggested that anal involvement generally occurs in the setting of extensive involvement of PV at other sites. Hotz et al. [15] studied 103 PV patients; seventeen of them had anal involvement. Signs in eleven patients included 9 cases of erosions, one pseudofissure, and one bulla. Regarding the association of anal involvement with the severity of PV, the inclusion of involvement of other mucosa including anal mucosa in any future criteria of severity of PV may be justified.

As expected, erosions were the most common anal sign. Pseudofissure—a linear erosion or ulcer not in a typical site for anal fissure (12 o'clock)—was also a frequent, noticeable finding. Hemorrhoids were found in 17 patients (10%) in anal examination. Interestingly, 16 out of 17 patients with hemorrhoids had anal PV; all of these 16 patients had erosions as the anal sign in their examination. Hemorrhoids are subject to repeated trauma during defecation, and it seems that they could be considered a vulnerable site for erosions in PV. Seventeen patients (10%) showed leukoedema described as pearly white appearance of the mucosa surrounding anal erosions. This has been previously described by Hotz et al. [15].

In conclusion, anal involvement in PV seems to be more frequent than previously assumed. Although most patients with anal lesions were symptomatic and had defecation problems, routine anal examination is recommended even in asymptomatic patients as it appears to correlate with the severity of PV.

## Figures and Tables

**Table 1:** Characteristics of pemphigus vulgaris patients with or without anal involvement.

	Total (*n* = 168)	Patients with anal involvement (*n* = 47)	Patients without anal involvement (*n* = 121)	*P* value for differences between patients with and without anal involvement
Age y; mean ± SD	44 ± 12.6	45 ± 13.3	44 ± 12.3	0.6
M : F ratio	1 : 1.21	1 : 0.96	1 : 1.33	0.4
Phenotype-*n* (%)				
Mucosal	8 (4.8)	2 (4.3)	4 (3.3)	0.08
Mucocutaneous	141 (84)	43 (91.5)	98 (81)	
Cutaneous	19 (11.2)	2 (4.3)	19 (15.7)	
Number (%) of patients with				
Oral involvement	137 (81.5)	41 (87.2)	96 (79.3)	0.24
Genital involvement	44 (26.2)	23 (48)	20 (16.5)	<0.001
Nasal mucosal involvement	38 (22.6)	17 (34)	22 (18)	0.02
Ophthalmic involvement	36 (21.4)	15 (32)	20 (16.5)	0.03

SD: standard deviation; M: male; F: female; *P* value less than 0.05 was considered significant.

**Table 2:** Symptoms and signs in patients with pemphigus vulgaris.

	Number (%) among total patients	% among patients with anal involvement
Symptoms		
Spontaneous pain	10 (6)	23.8
Bleeding	16 (9.5)	38
Pain on defecation	21 (12.5)	50
Constipation	31 (18.5)	73.8
Total	**42 (25)**	
Signs related to PV		
Erosion	43 (25.6)	91.5
Leukoedema	16 (9)	34
Perianal involvement	8 (4.8)	17
Pseudofissure	7 (4.2)	15
Ulcer	6 (3.5)	12.8
Vegetant	2 (1.2)	4.3
Bullae	1 (0.6)	2.1
Total	**47 (27.9)**	
Signs unrelated to PV		
Hemorrhoid	17 (10)	16 (34%)*
Prolapse	1 (0.6)	0**

*One of the patients shows hemorrhoid in absence of other anal pathology in physical examination.

**One patient showed prolapse in examination without any other anal pathology.
